# The psychological impact of COVID-19 pandemic and associated factors among college and university students in Ethiopia: a systematic review and meta-analysis, 2022

**DOI:** 10.3389/fpubh.2023.1136031

**Published:** 2023-07-13

**Authors:** Rahel Mulatie Anteneh, Anteneh Mengist Dessie, Melkalem Mamuye Azanaw, Denekew Tenaw Anley, Biruk Demissie Melese, Sefineh Fenta Feleke, Tiruayehu Getinet Abebe, Achenef Asmamaw Muche

**Affiliations:** ^1^Department of Public Health, College of Health Science, Debre Tabor University, Debre Tabor, Ethiopia; ^2^Department Public Health, College of Health Science, Woldia University, Woldia, Ethiopia; ^3^Department of Epidemiology and Biostatistics, Institute Public Health, College of Medicine and Health Science, University of Gondar, Gondar, Ethiopia

**Keywords:** COVID-19, prevalence, associated factors, psychological impact, Ethiopia

## Abstract

**Background:**

The Corona virus disease 19 (COVID-19) pandemic is a human tragedy that occurred in this era. It poses an unprecedented psychological, social, economic, and health crisis. The mental health and well-being of entire societies are suffering as a result of this crisis, but the suffering is greater in students at all levels of education and must be addressed immediately. Thus, this study was aimed to estimate the pooled prevalence and associated factors of the psychological impact of COVID-19 among higher education students.

**Methods:**

The potential studies were searched via PubMed, HINARI, the Cochrane Library, and Google Scholar. Studies were appraised using the Joanna Briggs Institute appraisal checklist. Micro Soft Excel was used to extract the data, which was then exported to Stata version 14 for analysis. Heterogeneity between studies was tested using Cochrane statistics and the *I*^2^ test, and small-study effects were checked using Egger’s statistical test. A random-effects model was employed to estimate the pooled prevalence of the psychological impact of COVID-19 and its associated factor.

**Results:**

After reviewing 227 studies, eight fulfilled the inclusion criteria and were included in the meta-analysis. The pooled prevalence of the psychological impact of Corona virus disease 19 among higher education students in Ethiopia, including depression, anxiety, and stress was 43.49% (95% CI: 29.59, 57.40%), 46.27% (95% CI: 32.77, 59.78%), and 31.43% (95% CI: 22.71, 40.15), respectively. Having a medical illness, being an urban resident, living with parents, having relative death due to pandemics, and having a non-health field of study were identified as significant associated factors for the impact of the pandemic in higher education students.

**Conclusion:**

The COVID-19 pandemic had a significant psychological impact on college and university students. Depression, anxiety, and stress were the most commonly reported psychological impacts across studies among higher education students. Hence, applying tele-psychotherapy using, smartphones, and social media platforms has an effect on reducing the impact. Programs for preventing and controlling epidemics should be developed by the government and higher education institutions that incorporate mental health interventions and build resilience.

## Introduction

The novel Severe Acute Respiratory Syndrome Coronavirus 2 (SARS-CoV-2), also called COVID-19, arose in Wuhan, China, at the end of 2019, and poses a global health threat (1). The COVID-19 outbreak was spreading rapidly not only in China, but also worldwide, therefore, the World Health Organization (WHO) announced it as an outbreak of a new coronavirus disease on January 30, 2020, and a pandemic on March 12, 2020 (2). The epidemic of COVID-19 has been extensively affecting the living and life of individuals globally, more specifically after the statement of an international epidemic by the WHO (3).

The infectious disease of the COVID-19 pandemic affected all aspects of human life, including business, research, education, health, economy, sport, transportation, worship, social interactions, politics, governance, and entertainment in all populations, including patients, healthcare workers, the community, and students (4–6). Following its discovery, ongoing attempts are being made to put an end to the COVID-19 pandemic. In spite of several interventions, such as the distribution of different COVID-19 vaccinations in many nations, including Ethiopia, the majority of the populace refused to receive the shots. The COVID-19 pandemic causes the highest number of deaths and morbidity and has a huge economic, psychological, and social impact on the world (7, 8).

The pandemic has triggered a global health crisis and is a major public health emergency of international concern all over the world, which not only threatens the lives of people but also affects their mental health, such as major depressive disorder, fear, and stress (9).

In order to control the spread of the pandemic, many restricted local prevention policies, such as contact tracing and quarantine, staying home, lockdown, social and physical distance, and the closure of different facilities and services, had been taken, although they affected the normal lives of the people. Ethiopia has also implemented a number of prevention and control measures to stop the spread of COVID-19, including installing hand washing stations in public areas (such as banks, churches, mosques, and markets), setting up isolation facilities, and declaring a state of emergency across the country. Patients with proven or suspected COVID-19, medical staff, and even the general public were under a great deal of stress due to the COVID-19 pandemic’s rapid escalation and global spread, which was unresponsive to measures implemented and increased the risk of mental health issues (10).

For mental health services, this unprecedented catastrophe poses major challenges (4, 11). Due to the severe contagiousness of the pandemic, inherent scientific uncertainty, and stringent quarantine, all of these factors unavoidably increase patients’ fear and stigma, which makes it harder for them to get the help they need for effective and efficient medical care and psychological crisis intervention (12).

The pandemic’s effects on mental health have different degrees of impact on all populations, but those who live in socially deprived areas and those who work in critical positions are disproportionately impacted (13). Due to worries about their capacity to perform academically and succeed, as well as other problems like future careers and college social life, college and university students are among those who have been most negatively impacted by the COVID-19 pandemic (14). Higher education students around the world report showed that higher levels of anxiety, depressive moods, poor self-esteem, psychosomatic disorders, drug abuse, and suicidality when a pandemic is not present in comparison to the general population (15–17). The study in Pakistan showed that of university students are considered a vulnerable populace, and particular interventions and preventions are required to protect and improve their mental health and quality of life during the epidemic globally (15).

During this pandemic period, having mental and psychological problems leads to poor self-care practices, appetite, sleep, immunity status, and compliance with the instructions given by the healthcare provider that exposed them to infectious a etiology (18).

Due to the pandemic, mental health consequences increased by 1,000% in the United States during the lockdown (19), and it had also huge burden in our country Ethiopia (16). The quick propagation of the virus, greater access to information, and greater case fatality rate of this illness all contribute to the rise in unpleasant psychological effects (20). In order to deal with the effects of the condition on their physical and mental health, students might require additional resources and services. The psychological effects of COVID-19 that were most frequently studied and reported were stress, anxiety, and depression (16). Despite the fact, that many prevention and control strategies were implemented to slow the course of the disorders in Ethiopia.

Numerous studies have been done on the effects of the COVID-19 pandemic on higher education students’ mental health (16), but no systematic reviews or meta-analyses have been carried out in Ethiopia. A systematic study and meta-analysis were conducted to evaluate the combined prevalence of the psychological effects of the COVID-19 pandemic and its related components among higher education students (HES).

For the COVID-19 pandemic’s psychological effects to be improved, it is crucial to understand the prevalence and associated factors among college students. It also directs the areas of concentration and intervention measures for educational institutions and policymakers to lessen the effects of any other pandemic. Because of this, the purpose of this study was to perform a systematic review and meta-analysis to examine the psychological effects of the COVID-19 pandemic and its associated factors among students in higher education.

## Materials and methods

### Study design and setting

A systematic review and meta-analysis were conducted to estimate the pooled prevalence of psychological impact of COVID-19 pandemic and its associated factor higher education students in Ethiopia.

### Searching strategies and sources

The method used to conduct this systematic review and meta-analysis was the PRISMA-2020 protocol (21). Without a time, limit, several works of literature were searched in databases like PubMed, CINHAL, the Cochrane Library, and search engines including Google Scholar. All searches are only available in English. To avoid any duplication, the searched literature was imported into Endnote X9. Between March 6 and March 12, 2022, a literature search was done. All papers released up until March 12, 2022, were taken into account. To identify the articles, the search terms of “Coronavirus,” “COVID-19,” “2019-ncov,” “SARS-cov-2,” “mental illness,” “mental health problem,” “distress,” “anxiety,” “depression,” “depressive symptom,” “emotional stress,” “associated factor,” “risk factor,” “predictor,” “determinates,” and all the possible combinations of these keywords were used.

### Eligibility criteria

The entire texts of the published articles on COVID-19 prevalence and related determinants of psychological impact among college or university students in Ethiopia with an outcome of interest were included. Using **CoCoPop,** the database search was organized so that inclusion and exclusion criteria for prevalence studies could be declared for the condition (psychological effects of COVID-19), context (Ethiopia), and population (college and university students).

### Inclusion criteria and exclusion criteria

All studies conducted on the prevalence and associated factors of COVID-19’s psychological impact among college and university students in Ethiopia were included. Besides, all English-language full-text articles and all published articles were eligible to be included in this systematic review. Studies with no prevalence report on the psychological impact of COVID-19, unrelated research work, full text not available, and duplicate data sources were excluded.

### Study selection

All studies found in various databases were merged, exported, and managed using Endnote X9 software. The full text of every duplicate article that was regularly discovered in different databases was searched both manually and with Endnote software. The entire texts of the studies that survived the screening step were carefully checked in accordance with the criteria, and a number of other unrelated studies were also eliminated. All titles and abstracts found in the electronic databases were screened. Article review and data extraction tasks were carried out separately by two reviewers to avoid subjectivity. Whenever there was a difference of opinion among the three reviewers, when an article wasn’t included, the exclusion was explained.

### Data extraction

Using a data extraction checklist prepared and evaluated by all authors, data were taken from each of the journal articles included in the review. The articles that met the criteria for inclusion were extracted and put on a separate data sheet. The study design, various psychological impacts (depression, anxiety, and stress) with prevalence, the authors’ names, the years of publication, total sample size, the population under study, the proportion of sexes, the average respondent age, the study area, estimated prevalence, potential factors, and upper versus lower boundary of the estimated effect of factors are all listed on the data extraction tool.

### Quality assessment

The Joanna Briggs Institute (JBI) quality appraisal checklist used for cross-sectional research was utilized to evaluate each study’s quality (19). The critical evaluation checklist comprised nine parameters, and responses ranged from “yes,” “no,” “unclear,” and “not relevant.” The quality of each study was declared using the major assessment tools (methodological quality, comparability, and outcome and statistical analysis of the study). Two researchers independently evaluated the caliber of the studies that were included. When there were differences, they were settled through dialogue or bargaining with a third party. If a study received a quality assessment indicator score of 50% or higher, it was deemed as low risk.

### Statistical analysis and risk of bias

The retrieved information was entered into STATA version 16 statistical software after being exported from Microsoft Excel 2016. Narratives, tables, and figures were used to convey the descriptive summaries of the included studies, and prevalence and pooled odds ratios were also reported. The pooled odds ratio was calculated for the commonly associated risk factors of the reported studies. With Cochrane Q-statistics of 25, 50, and 75 percent, low, moderate, and severe heterogeneity, respectively, was determined for reported prevalence heterogeneity using the inverse variance (*I*^2^) and a *p*-value less than 0.05 (20). The forest plot was also used to show the presence of heterogeneity (21). A sub-group analysis was performed to identify the possible source of heterogeneity. Furthermore, sensitivity analysis was conducted to determine the effect of single studies on the pooled estimate. The combined prevalence of COVID-19’s psychological effects and their contributing components were calculated using a random effect meta-analysis method (21). Publication bias (the small study effect) was detected using funnel plot symmetry, and the statistical significance was assessed using both Egger’s test Egger et al. (21) and the Beggar statistical test.

## Results

### Search results and study selection

**Figure 1:**
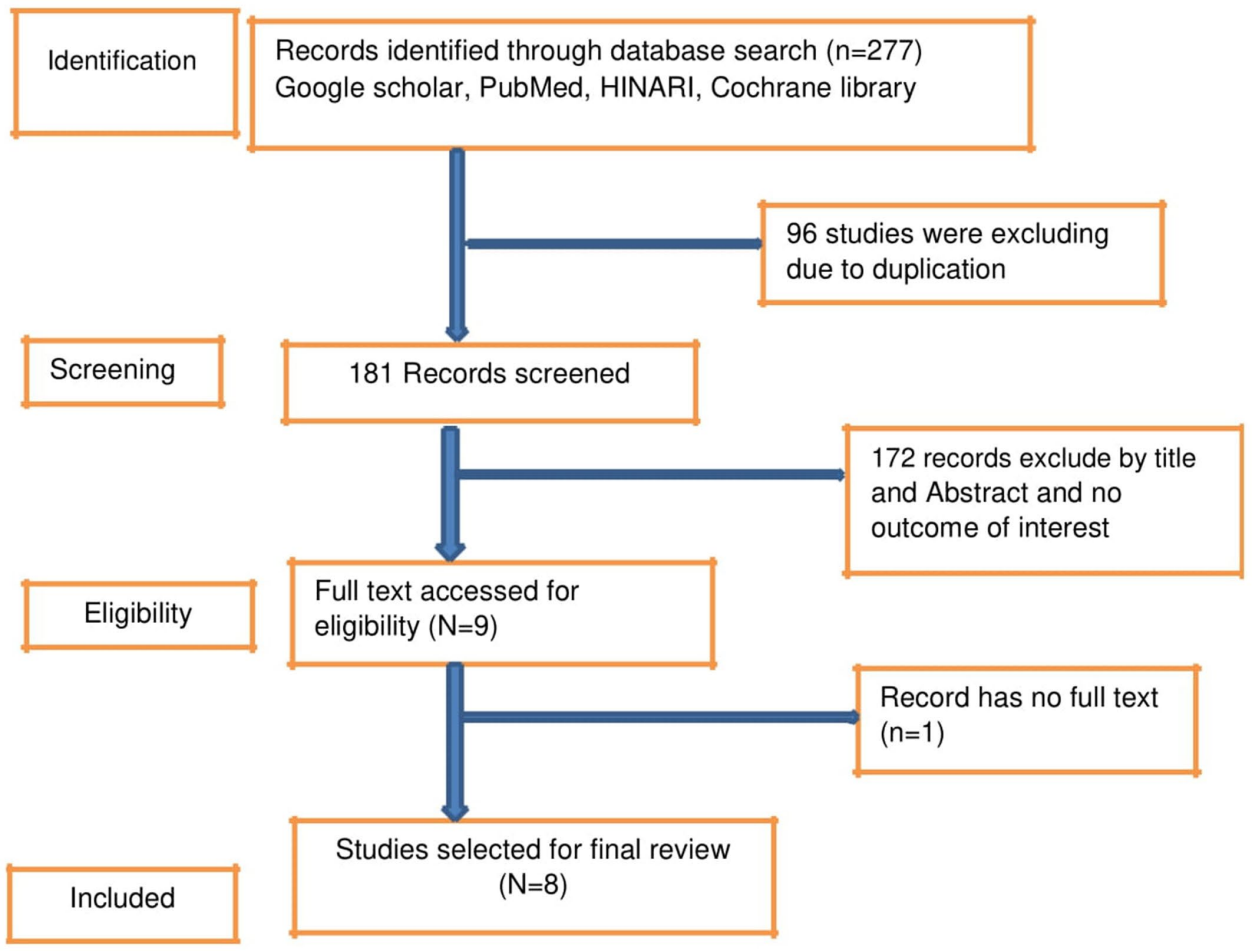
Flow chart of the systematic research and study selection process.

A total of 227 records were retrieved using different databases such as PubMed, Google Scholar, Hinari, and Cochrane Library, which were exported to Endnote X9. After importing all the identified articles to EndNote X9, 96 studies were excluded due to duplication. Then, 181 studies were screened for title and abstract, and 173 papers were removed due to unrelated titles and not reporting the outcome of interest. Finally, the full text of eight eligible studies was reviewed (Figure 1).

### Characteristics of studies included in this review

A total of eight articles were included in this systematic review and meta-analysis. All the included articles were published in 2020 and 2022. All studies employed a cross-sectional study design, of which 2 were community-based, 4 were web-based, and the remaining 2 were institutional-based. A total of 3,489 higher education students participated in these studies using an estimated sample size ranging from 153 (22) up to 779 (23) (Table 1).

**Table 1:** Characteristics of 8 studies included to estimate the pooled prevalence of psychological impact of COVID-19 and associated factors among HES in Ethiopia.

Author name	Study design	Study setting	Study population	Sample size	Psychological impact	Prevalence (%)	Quality
Abay W. Tadesse	Cross sectional	Dessie	College students	408	Depression	77	Low risk
					Anxiety	71.8	
					Stress	48.5	
Addisu Tadesse	Web based cross sectional	Addis Abeba	College students	153	Depression	51	Low risk
					Anxiety	51.6	
					Stress	11	
Enyew Getaneh	Institution based cross sectional	University Of Gondar	University students	338	Depression	40.2	Low risk
					Anxiety	39.6	
					Stress	22.2	
Mengistu Awoke	Cross sectional	Jimma	University students	337	Stress	35.9	Low risk
Mesfn Esayas Lelish	Web based cross sectional	Mizan tepi	University students	779	Depression	39.5	Low risk
Nigusie Shifera	Community based cross sectional	Bench-shiko	University students	314	Depression	21.2	Low risk
					Anxiety	52	
					Stress	28.8	
Wudneh Simegn	Web based cross sectional	Ethiopia	University students	423	Depression	46.3	Low risk
					Anxiety	52	
					Stress	28.8	
Zebene M. Assefa	Institution based cross sectional	Wolkite	University students	710	Depression	30	Low risk
					Anxiety	35.1	
					Stress	38	

### Prevalence of psychological impact of COVID-19

**Figure 2:**
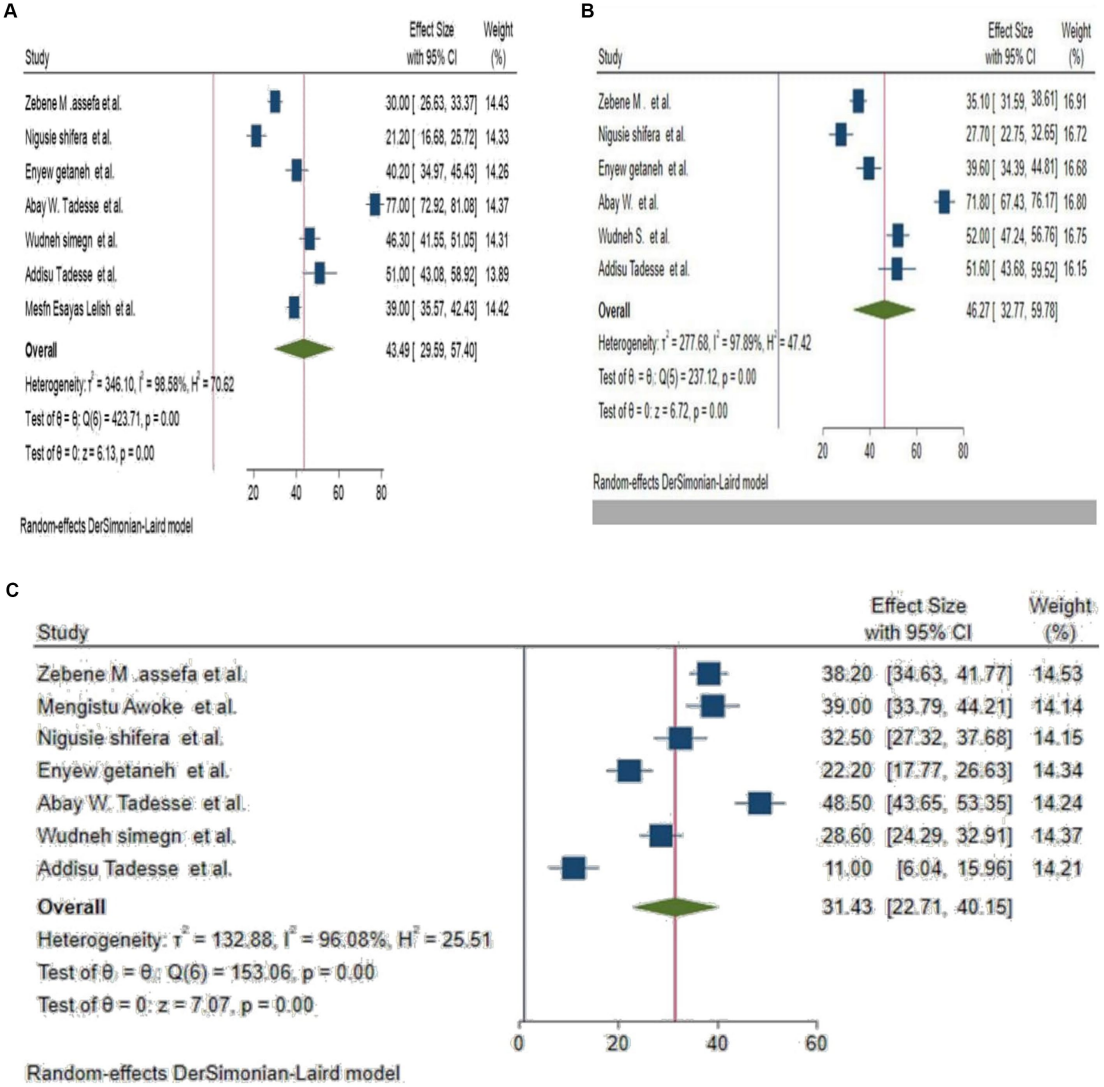
**(A–C)** Forest plot for pooled prevalence depression, anxiety and stress among HES, respectively.

Using a DerSimonian and Laird random-effects model, the overall pooled prevalence of the psychological impact of COVID-19 among higher education students in Ethiopia was depression 43.49% (95% CI: 29.59, 57.40%), anxiety 46.27% (95% CI: 32.77, 59.78%), and stress was 31.43% (95% CI: 22.71, 40.15), with significant heterogeneity between studies (*I*^2^ = 98.58, 97.89, and 96.08%, *p* < 0.001), respectively. The overall pooled prevalence of the psychological impact of COVID-19 among HES in Ethiopia was presented using a forest plot for depression, anxiety, and stress, respectively (Figure 2).

### Heterogeneity and publication bias

The Cochrane test and the *I*^2^ test were used to assess heterogeneity. *I*^2^ values in this meta-analysis for depression, anxiety, and stress were *I*^2^ = 98.58, 97.89, and 96.08%, *p* < 0.001, respectively, indicating that there was heterogeneity. Subgroup and sensitivity analyses were used to further explore it. According to a subgroup analysis based on the study population, the prevalence of the depression effect of COVID-19 on college students is higher [64.23% (95% CI: 38.75, 89.70%)] than that on university students [35.29% (95% CI: 27.29, 43.3%)].

The prevalence of anxiety was in sub group analysis using study population was also higher in college students [61.98, 95% CI: 42.19%, 81.77%] than university students [38.58% (95% CI: 29.06, 48.1%)]. And the prevalence of Stress was lower in college students [29.754% (95% CI: 26.99, 56.5%)] than university students’ [32.087 (95% CI: 25.79, 38.37%)].

**Figure 3:**
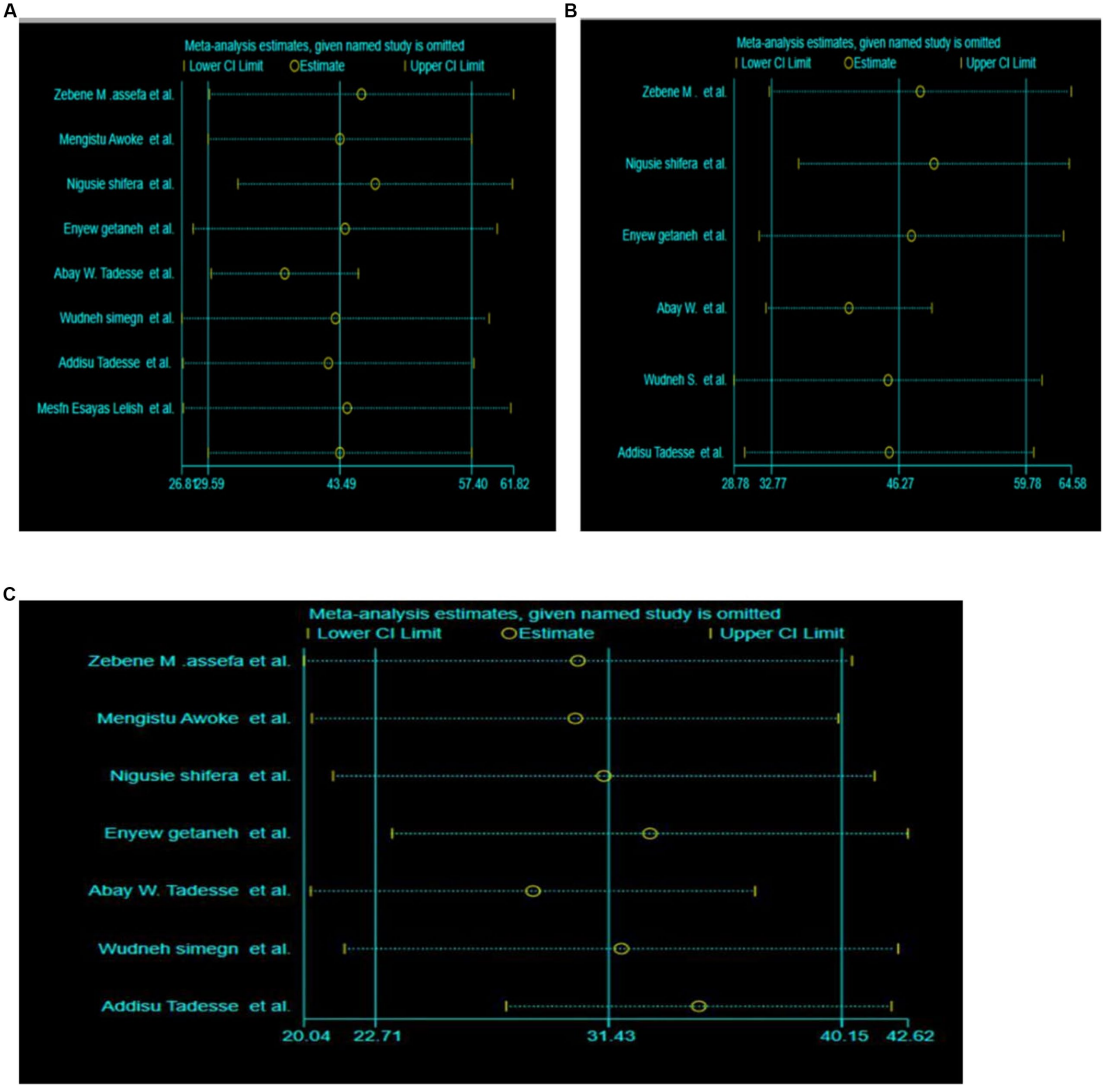
**(A–C)** Sensitivity analysis of the pooled prevalence of depression, anxiety and stress effect of COVID-19 among HES, respectively.

Sensitivity analysis was also used to look into the impact of a single study on the overall magnitude estimate, with the results indicating that a single study did not have a significant impact on the overall magnitude estimate. As a result, the point estimate of its omitted analysis falls within the combined analysis confidence interval (Figure 3).

### Publication bias

**Figure 4:**
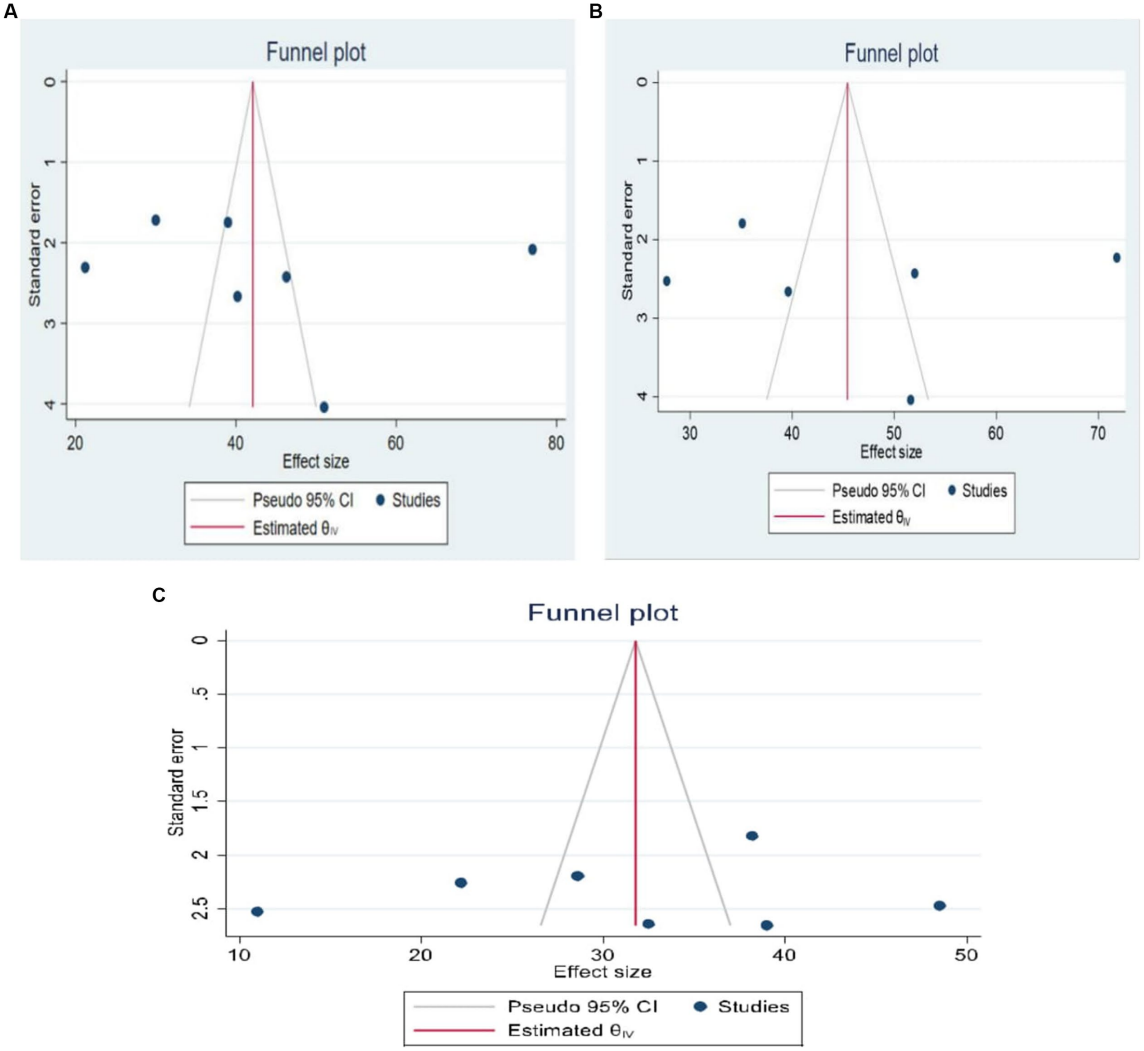
**(A–C)** Funnel plot test for publication bias for depression, anxiety and stress, respectively.

Using funnel plots and objective assessments (Egger’s test) the presence of publication bias was investigated. Funnel plots assessing the risk of publication bias showed symmetrical distribution, which was confirmed by the Egger tests, which yielded a *p*-value >0.05 (Figure 4).

### Factors associated with psychological impact of COVID-19

#### Factors associated with depression

Factors including insomnia, medical illness, residence, the field of study, living with family, sex, availability of protective equipment, and relative illness or death with COVID-19 were associated with depression.

Students who had a sleeping disorder (insomnia) had a 1.72-times (95% CI: 1.33–2.26) higher risk of developing depression than those who did not have insomnia, and students who had a medical illness had a 3.25-times (95% CI: 1.9–5.4) higher risk of developing depression than those who did not have a medical illness and a relative had developed depression. COVID-19 had a 2.43-fold (OR = 2.43, 95% CI: 1.46–4.02) higher risk of developing depression compared with those who had not. Students from urban residences had a protective effect on depression, which decreased by 36.4% (OR = 0.636, 95% CI: 0.422–0.96) relative to those from rural residences (Table 2).

**Table 2:** Summary of the pooled effects of factors associated with psychological impact of COVID-19 in Ethiopia.

Variable (non-reference group)	Pooled odds ratio with 95% CI		
	Depression	Anxiety	Stress
Sex (female)	1.25 (0.5, 3.13)	1.26 (0.67, 2.36)	1.21 (0.43, 3.4)
Having protective equipment (no)	1.85 (0.335, 9.6)	1.03 (0.354, 3.01)	
Substance use (yes)	1.3 (0.987, 1.87)	1.4 (0.24, 8.256)	
Live with parent (no)	2.2 (0.9, 5.1)	**2.4 (1.6, 3.5)**	0.7 (0.16, 2.9)
Insomnia (yes)	**1.72 (1.3, 2.2)**		
History of mental illness (yes)			1.044 (0.246, 4.40)
Residence (urban)	**0.63 (0.4, 0.96)**	**0.57 (0.42, 0.81)**	**0.65 (0.45. 0.92)**
Field of study (non-health)	1.18 (0.1, 1.29)	**3.27 (1.39, 7.50)**	**3.95 (2.5, 6.5)**
Relative got COVID-19 (yes)	**2.42 (1.46, 4.02)**	1.98 (0.69, 5.72)	
Medical illness (yes)	**3.25 (1.9, 5.4)**	**2.49 (1.50, 4.1)**	**2.47 (1.48, 4.12)**

Bold = significant at *p*-value < 0.05.

#### Factors associated with anxiety

According to our pooled data, students not living with their parents had a 2.4-fold (OR = 2.40, 95% CI: 1.6–3.56) higher risk of developing anxiety compared to those living with their parents. In addition, having a medical illness was linked to a 2.5-fold (OR = 2.5, 95% CI: 1.5–4.1) increased risk of developing anxiety when compared to not having a medical illness.

When compared to students in the health field, students in the non-health field had a 3.2-fold (OR = 3.227, 95% CI: 1.39–7.5) higher risk of developing anxiety. Students from urban areas were less likely to develop anxiety than those from rural areas (coefficient of variation: 0.58; 95% CI: 0.354–0.82; Table 2).

#### Factors associated with stress

As a result, our pooled data showed that students from urban residences were 33.5% (OR = 0.645, 95% CI: 0.45–0.923) less likely to develop stress than those from rural residences. Students who had medical illnesses were 2.47 times more likely to experience stress (OR = 2.47, 95% CI: 1.4–4.5) than those who did not have medical illnesses. Students in the non-health field of study were four times (OR = 4.0, 95% CI: 2.5–6.5) more at risk for stress compared with those in the health field. Students who were female were (OR = 1.21, 95% CI: 0.43, 3.4) and had a history of mental illness (OR = 1.044, 95% CI: 0.246, 4.40) increase the risk of stress psychological impact in contrast to the comparison group, but the effect was not significant (Table 2).

## Discussion

This study evaluated the pooled prevalence and combined impact of factors related to the pandemic’s psychological effects on HES in Ethiopia. According to our findings, the pooled prevalence of depression, anxiety, and stress during the COVID-19 pandemic among higher education students in Ethiopia was 43.49%, 46.27%, and 31.43%, respectively. Recent studies have similarly shown that COVID-19 affects mental health outcomes such as anxiety, depression, and post-traumatic stress symptoms (24, 25).

Based on our pooled estimate anxiety is the most prevalent psychological disorder followed by depression. This finding was in line with study from Pakistan (15, 26). The pooled prevalence of Anxiety among higher education students in our finding was higher than study conducted in Chinese student 26% (95% CI, 21%–30%) (27), but in line with systematic review meta-analysis findings from Spain 28% (95% CI: 22%–34%) (28), Canada 32% (29), Iran 31.9% (95% CI: 27.5–36.7) (24), China among non-Chinese students 36% (95% CI, 26%–46%) (27). But lower in Bangladesh 87.7% (30) and Egypt 70.5% (31).

Depression is a common psychological state affecting many people from all age groups and key role in worsening the prognosis of chronic diseases (32, 33). The pooled prevalence of depression in Ethiopia was 43.49%, which is comparable to the reports from China 37% (95% CI, 32%–42%) (27), Canada 34% (29), Iran 33.7% (95% CI: 27.5–40.6) (24), and from global pooled report 34% (29). However, it was higher than another study conducted in china 26% (23.3, 28.5) (34), but lower than Bangladesh 82.4% (30).

The pooled prevalence of stress was comparable with findings from India 29.6% (95% CI: 24.3–35.4) (35), and China 23% (95% CI, 8–39) (27). However, it was lower than studies from Europe 62% (41, 79%) (36), and Brazilian 57.5% (37). The possible reason for the discrepancy might be due differences in strict quarantine, incidence rate, the effect of lockdown, the difference in literacy level, study sittings difference, and environmental factor.

Factors associated with depression included a relative having COVID-19, insomnia, medical illness, and residence, all of which had a significant effect on the expression of depression symptoms in higher education students. Our pooled effect shows that living in an urban area reduces the risk of depression by 37% among higher education students; this finding is supported by a global systematic and meta-analysis report (38), United States (4), and China (39). In addition, it is supported by study finding from Gondar, Ethiopia (40). In contrast to this finding, a study conducted in Bangladesh (30) showed that being urban residence was a risk factor for having depression among students. Having medical condition increased the chance of depression among HES by 3.2 times compared to not having one. The study was consistent with a conclusion corroborated by a thorough analysis of the COVID-19 pandemic’s effects on mental health among medical students (28). This study also supported by findings from China (34) and Brazil (37). This is because having a past medical history may make COVID-19 effects worse, and comorbidities make COVID-19 effects more severe and fatal.

Students who had a medical illness was 3.2 times higher risk of depression than those who had not a medical illness. The finding was supported by other studies regarding COVID-19 pandemic and mental health consequences (31, 41). This is due to the fact that having a previous history of medical illness may exacerbate the mental impacts of COVID-19, its severity, and fatality of the disease. When compared to the comparable group, students with insomnia and COVID-19 relative death/illness have depression risks that are 1.7 and 2.4 times higher, respectively. Factors such as residence, medical illness, and field of the study showed a significant effect on the development of anxiety and stress.

Having a medical illness is a significant factor that increase the risk for the psychological impacts of COVID-19 among HES, whereas students were from urban residences had reduce effect on the disease’s psychological impact as compared with the counterpart. This finding was consistent with findings from China (39), united states (42). Students from the non-health department (field of study) were 3.2 and 4 times risk for anxiety and stress as compared with the health field of study, respectively. This was consistent with a systematic review findings done based on data from countries including China, Spain, Italy, Iran, the US, Turkey, Nepal, and Denmark (38). The reason might be due to the fact that medical students exhibit high levels of resilience, which favorably correlate with effective problem-solving techniques or adaptive coping strategies when facing a problem (43–45).

### Strength and limitations

The strength of this study includes the use of multiple databases to search articles (both manually and electronically) for meta-analysis and the abstraction of information uniformly using a predetermined and pretested standard format by two independent reviewers that helped to minimize error. This meta-analysis also included studies from different parts of the country among both college and university students. Despite their strength, there were some potential limitations to those studies. These limitations include the fact that they are all cross-sectional articles written in the English language. Additionally, there is substantial heterogeneity. Furthermore, because the studies relied on self-reported data, the prevalence of COVID-19 could have been overestimated or underestimated due to the social desirability bias.

## Conclusion and recommendation

The pooled proportion of psychological impact from COVID-19 among higher education students in Ethiopia was high. The most commonly reported psychological impacts were anxiety and depression. Insomnia, a medical condition, place of residence, and a family member contracting COVID-19 or passing away from it were all major predictors of depression. Non-health field of study, living with a parent, urban resident, and having medical illness were significant factors for anxiety. Living with parents and having a medical condition are significant predictors of the stress psychological effects of COVID-19. The results can be used to quantify the support requirements of students and to inform tiered and customized pandemic interventions that increase resilience and reduce vulnerability. This contributes to improving motivation for quick action. Therefore, it is crucial to provide psychological therapy, establish coping mechanisms, and address other issues in order to minimize the COVID-19 pandemic’s negative effects on mental health. Disease infectivity and fatality rates are also continuing to rise across the nation.

Governmental and private organizations and healthcare providers also provide psychosocial and mental health services alongside healthcare services and various media channels, front line health workers, social media platforms, email, and electronic letters to promote psychological support. Moreover, the government should incorporate mental health and psychological intervention within any outbreak prevention and mitigation program.

## Data availability statement

The original contributions presented in the study are included in the article/Supplementary material, further inquiries can be directed to the corresponding author.

## Author contributions

RA contributed to study conception and design, data analysis, interpretation, and manuscript drafting. AD, MA, and TA were involved in, literature searching data analysis, manuscript preparation, tables and figures, and critical revision. BM, SF, DA, and AM contributed to the literature search, data analysis, interpretation, and manuscript writing. BM had significant contributed on this manuscript starting from conceptualization, till literature searching, date extraction, analysis & interpretation, manuscript preparation, and revision of the manuscript. All authors were involved in reviewing the final draft of the manuscript, revision of the manuscript, and approved the submitted version.

## Conflict of interest

The authors declare that the research was conducted in the absence of any commercial or financial relationships that could be construed as a potential conflict of interest.

## Publisher’s note

All claims expressed in this article are solely those of the authors and do not necessarily represent those of their affiliated organizations, or those of the publisher, the editors and the reviewers. Any product that may be evaluated in this article, or claim that may be made by its manufacturer, is not guaranteed or endorsed by the publisher.

## Abbreviations

POR: Pooled Odds Ratio
CI: Confidence Interval
HES: Higher Education Students
COVID-19: Coronavirus Disease of 2019
WHO: World Health Organization
